# Protective Role of *Psoralea corylifolia* L. Seed Extract against Hepatic Mitochondrial Dysfunction Induced by Oxidative Stress or Aging

**DOI:** 10.1155/2013/678028

**Published:** 2013-08-29

**Authors:** Eunhui Seo, Yoon Sin Oh, Donghee Kim, Mi-Young Lee, Sungwook Chae, Hee-Sook Jun

**Affiliations:** ^1^College of Pharmacy and Gachon Institute of Pharmaceutical Science, Gachon University, Incheon 406-840, Republic of Korea; ^2^Lee Gil Ya Cancer and Diabetes Institute, Gachon University, Incheon 406-840, Republic of Korea; ^3^Gachon Medical Research Institute, Gil Hospital, Incheon 405-760, Republic of Korea; ^4^KM-Based Herbal Drug Research Group, Korea Institute of Oriental Medicine, Daejeon 305-811, Republic of Korea

## Abstract

The accumulation of oxidative damage and mitochondrial dysfunction is an important factor that contributes to aging. The *Psoralea corylifolia *seeds (PCS), commonly known as “Boh-Gol-Zhee” in Korea, have been used traditionally as a medicinal remedy. We investigated whether an extract of PCS has protective effects on oxidative stress and mitochondrial function in hepatocytes. The PCS extract showed an antisenescence effect on human diploid fibroblasts as evidenced by a decreased expression of p16^INK4a^ mRNA and senescence-associated **β**-galactosidase staining. PCS extract treatment reduced H_2_O_2_-induced reactive oxygen species (ROS) production in HepG2 cells, inhibited ROS production in hepatocytes of aged mice, and increased superoxide dismutase activity. In H_2_O_2_-treated HepG2 cells, PCS extract treatment recovered ATP production. PCS extract treatment recovered the oxygen consumption rate and inhibited reduction of mitochondrial membrane potential induced by oxidative stress, suggesting improvement of mitochondrial function. In addition, PCS extract treatment recovered peroxisome proliferator-activated receptor **γ** coactivator 1**α** and carnitine palmitoyltransferase 1 mRNA and protein expression, and inhibited mitochondrial genome damage. Treatment with the major component of PCS extract, bakuchiol, also recovered mitochondrial dysfunction. On the basis of these results, we conclude that PCS extract inhibits ROS production and mitochondrial dysfunction induced by oxidative stress in hepatocytes.

## 1. Introduction

Oxidative stress is the imbalance between the production of reactive oxygen species (ROS) and a biological system's ability to readily detoxify the reactive intermediates or easily repair the resulting damage [1]. Disorders in the normal redox state of cells can cause toxic effects through the production of ROS, which include free radicals and peroxides [2]. In humans, oxidative stress is involved in many diseases and may exacerbate their symptoms [3]. As well, aging is known to have a close relationship with ROS [4]. 

The free-radical theory of aging suggests that many age-related pathologies result from damage to macromolecules by ROS [5, 6]. Mitochondria are the major source and target of ROS [7]. During aging, mitochondria lose their function, the number of mitochondria decreases, and ATP production declines [7–10]. Thus, antioxidant therapy and functional recovery of mitochondria may serve as a treatment approach for inhibiting oxidative stress and aging-associated diseases.

There is a growing interest in plant-based dietary components to counteract oxidative stress-induced disease. The seeds of *Psoralea corylifolia*, commonly known as “Boh-Gol-Zhee” in Korea, have been used traditionally as a medicinal remedy. Six compounds, bakuchiol, psoralen, isopsoralen, corylifolin, corylin, and psoralidin, are the major components of *Psoralea corylifolia* seed (PCS) extract. Among them, bakuchiol, a polyphenol compound, has been the most commonly studied. PCS extract is used in a variety of diseases such as leucoderma [11] and for impotence [12] and has antitumor [13] and antibacterial effects [14, 15]. In particular, PCS extract and bakuchiol have been reported to have a protective effect on hepatic injury [16, 17]. However, the mechanism of action is not fully understood. In this study, we examined whether PCS extract has an antioxidant effect and improves mitochondrial function in hepatocytes, as hepatocytes are exposed to large amounts of ROS due to their numerous mitochondria and high respiratory rate.

## 2. Materials and Methods

### 2.1. Materials

Dulbecco's Modified Eagle's Medium (DMEM) and FBS were purchased from Gibco BRL (Grand Island, NY). Fluorescein di-*β*-D-galactopyranoside was purchased from Molecular Probe (Eugene, OR). Antibodies against catalase, glutathione peroxidase 1/2 (GPX 1/2), superoxide dismutase (SOD) 1, SOD2, and carnitine palmitoyltransferase 1 (CPT1) were obtained from Santa Cruz Biotechnology Inc. (Santa Cruz, CA). Antibodies against actin and peroxisome proliferator-activated receptor *γ* coactivator 1*α* (PGC1*α*) were obtained from Sigma-Aldrich (St. Louis, MO) and Abcam (Cambridge, MA), respectively. Bakuchiol was purchased from Enzo Life Sciences Inc. (Farmingdale, NY). Resveratrol was obtained from Sigma-Aldrich and was used as a positive control. 

### 2.2. Preparation of PCS Extract

The PCS used in the present study was purchased from an oriental drug store (Kwang Myung Dang Co., Ulsan, Korea), and the voucher specimen was deposited in the Herbarium of Korea Institute of Oriental Medicine (KIOM) under registration number KIOM-111930. The extract was prepared by the standard procedure. In brief, the dried seeds (300 g) were ground into small pieces and then extracted with distilled water under reflux two times. The combined water extract was evaporated *in vacuo* to give a dark brownish residue (61.92 g).

### 2.3. Primary Hepatocyte Isolation

C57BL/6 male mice (Korea Research Institute of Bioscience and Biotechnology, Daejeon, Korea) were anesthetized, and their livers were perfused with 142 mM NaCl, 6.7 mM KCl, 10 mM HEPES, 2.5 mM EGTA, and pH 7.4. This solution was replaced by 0.5 mg/mL collagenase and 10 mg/mL albumin in 66.7 mM NaCl, 6.7 mM KCl, 10 mM HEPES, 4.8 mM CaCl, and pH 7.6. The perfused livers were removed, rinsed, and disaggregated. After centrifugation, cells were suspended in an appropriate volume of the culture medium (Hepatozyme-SFM, Gibco-BRL).

### 2.4. Cell Culture

Human diploid fibroblasts (HDF) were obtained from Dr. S.C. Park, Gachon University [18]. HDF and HepG2 cells (ATCC, Rockville, MD) were maintained at subconfluence at 37°C with 5% CO_2_. The cells were grown in DMEM with 10% FBS containing 100 units/mL of penicillin and streptomycin.

### 2.5. Cell Viability Assay

HDF cells (1 × 10^4^ cells/well) were grown in 96-well plates for 24, 48, or 72 h with 100 *μ*g/mL PCS extract. A Cell Counting Kit-8 (CCK-8) (Dojindo Laboratories, Kumamoto, Japan) was used to measure cell viability.

### 2.6. Senescence-Associated *β*-Galactosidase (SA-*β*-gal) Assay

HDF cells (5 × 10^3^ cells/well) were cultured in 96-well plates overnight for attachment. Cells were treated with PCS extract (50 *μ*g/mL) for 72 h and then incubated for 48 h in normal media. SA-*β*-gal staining was performed using a Senescence Detection Kit (Bio Vision, Mountain View, CA) using fluorescein di-*β*-D-galactopyranoside [19].

### 2.7. Quantitative Real-Time RT-PCR (qRT-PCR) Analysis

The total RNA was extracted from the cultured cells using TRIzol reagent (Invitrogen Corp., Carlsbad, CA, USA), following the manufacturer's instructions, and cDNA was synthesized using a PrimeScript 1st strand cDNA synthesis kit (Takara Bio Inc., Kyoto, Japan). qRT-PCR was performed using the SYBR Premix Ex Taq II, ROX plus (Takara Bio Inc.) and the Prism 7900HT sequence detection system (Applied Biosystems, Foster City, CA). PCR was carried out for 40 cycles (2 minutes at 50°, 10 minutes at 95°, and 40 cycles of 10 seconds at 95° and 1 minute at 60°). The primer sequences used are shown in Table 1. The relative copy number was calculated using the threshold crossing point (Ct) as calculated by ΔΔCt.

### 2.8. ROS Detection

For quantification of intracellular ROS levels, cells were loaded with 10 *μ*M 2′,7′-dichlorodihydrofluorescein diacetate (H_2_DCFDA; Molecular Probes) for 30 min at 37°C, 5% CO_2_ in phosphate-buffered saline (PBS). Cells were collected, washed twice with PBS, and suspended in 500 *μ*L PBS. Fluorescent intensity was measured using FACS Calibur (BD Biosciences, San Jose, CA) and analysed by CellQuest Pro 5.2 according to the manufacturer's protocol.

### 2.9. Western Blotting

Cells were solubilized with Mammalian Protein Extraction Buffer (GE Healthcare, Milwaukee, WI) containing protease inhibitor cocktail (Sigma-Aldrich). Proteins (30–50 *μ*g) were resolved by 8 or 15% sodium dodecyl sulfate polyacrylamide gel electrophoresis, transferred onto membranes, and blocked with tris-buffered saline containing Tween 20 in 5% nonfat dry milk. The membranes were incubated with specific primary antibodies and visualized by incubating with horseradish peroxidase-conjugated secondary antibodies. Chemiluminescence was detected by LAS-4000 (Fuji Film, Tokyo, Japan) after adding Immobilon Western Chemiluminescent HRP Substrate (Millipore, St. Charles, MO). 

### 2.10. SOD and GPX Activity Measurements

SOD and GPX activities were determined using a Superoxide Dismutase Assay Kit and Glutathione Peroxidase Assay Kit, respectively, following the manufacturer's instructions (Cayman Chemical, Ann Arbor, MI).

### 2.11. ATP Level and ADT/ATP Ratio Measurements

ATP levels and the ADP/ATP ratio were measured using the ADP/ATP Ratio Assay Kit (Abcam).

### 2.12. Mitochondrial DNA Long PCR

Genomic DNA was isolated using a genomic DNA isolation kit (Bioneer, Daejon, Korea). To amplify half of the mitochondrial genome (8.7 kb), we used the Expand Long Template PCR System (Roche Applied Science, Mannheim, Germany). The long PCR was carried out with 500 ng genomic DNA in a 50 *μ*L final volume (Primers F: AAGGATCCTCTAGAGCCCACTGTAAAG, R: TTGGATCCAGTGCATACCGCCAAAAG). PCR products were run on 0.8% agarose gels.

### 2.13. Oxygen Consumption Rate (OCR)

HepG2 cells were plated at 1 × 10^4^ cells/well and cultured on Seahorse XF-24 plates (Seahorse Bioscience, Billerica, MA). After overnight incubation, PCS extract was added for 20 h. On the day of metabolic flux analysis, cells were changed to unbuffered DMEM (Seahorse Bioscience), and incubated at 37°C in a non-CO_2_ incubator for 1 h. OCR was automatically calculated and recorded by the Seahorse XF-24 analyzer (Seahorse Bioscience). 

### 2.14. Mitochondrial Membrane Potential Measurements

HepG2 cells were seeded 5 × 10^5^ cells per well in 6-well plates. After overnight incubation, cells were treated with PCS extract for 24 h, and 2 or 4 mM H_2_O_2_ was added and incubated for the last 6 h. The mitochondrial membrane potential was determined using a mitochondrial membrane potential assay kit (Biotium, Hayward, CA) and the BD LSR II flow cytometer (BD Biosciences).

### 2.15. Statistical Analyses

All data are expressed as mean ± standard error of at least three independent experiments. Data were analyzed using Analysis of Variance followed by post-hoc analysis using the Bonferroni test (SPSS 10.0 statistical software). *P* values less than 0.05 were considered statistically significant.

## 3. Results

### 3.1. Effects of PCS Extracts on Senescent Cells

**Figure 1:**
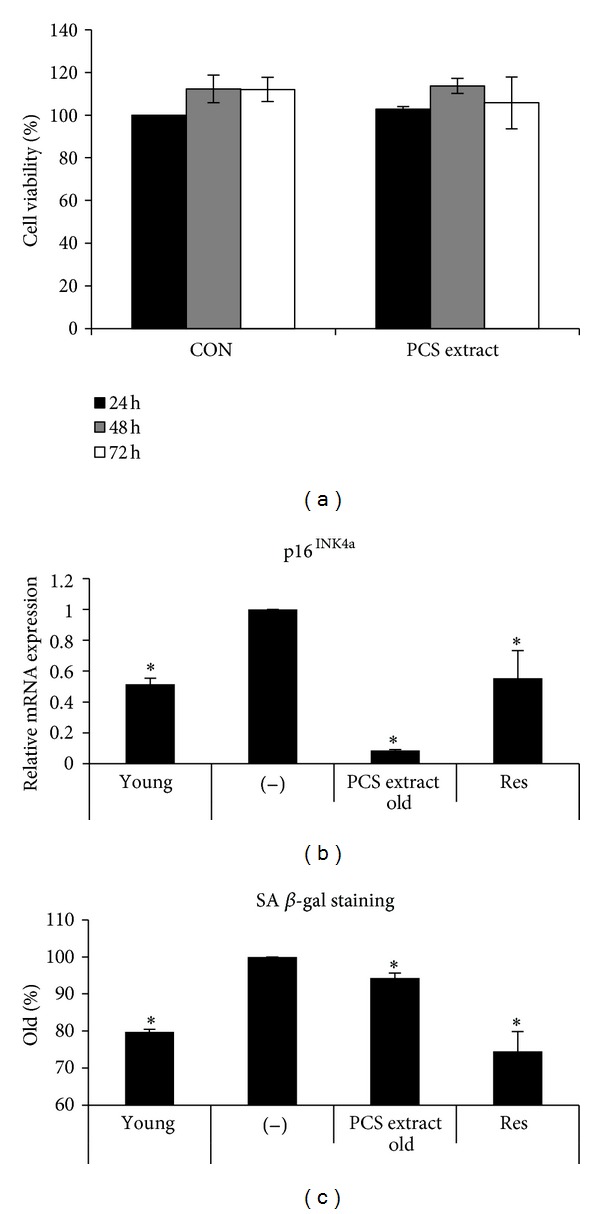
Effects of the PCS extract on senescent HDF cells. (a) HDF cells were treated with PCS extract (100 *μ*g/mL) or water as a control (CON) for 24, 48, or 72 h. Cell viability was measured by a CCK-8 assay kit. (b) HDF cells were treated without (−) or with the PCS extract (50 *μ*g/mL) for 24 h. mRNA for p16^INK4a^ was analyzed by qRT-PCR. (c) HDF cells were treated without (−) or with the PCS extract (50 *μ*g/mL) for 72 h and incubated for 48 h in normal media. SA-*β*-gal was detected using a Senescence Detection Kit. “Young,” less than 13 passages, “old,” more than 32 passages. Resveratrol (Res, 50 *μ*M) was used as a positive control. *P < 0.05 versus (−)/old HDF cells.

In order to determine whether PCS extracts have any toxicity in cells, we treated HDF cells with 100 *μ*g/mL of PCS extract for 24, 48, or 72 h. No cytotoxic effects were observed (Figure 1(a)).

To examine the effects of PCS extract in senescent cells, “Old” HDF cells (more than 32 passages) were treated with PCS extract (50 *μ*g/mL) for 24 h, and p16^INK4a^ mRNA expression was analyzed by qRT-PCR. “Young” HDF cells (less than 13 passages) were used for comparison. The expression of p16^INK4a^ mRNA and SA-*β*-gal-staining was significantly lower in young as compared with old untreated HDF cells. In old HDF cells, PCS extract treatment significantly reduced p16^INK4a^ mRNA and SA-*β*-gal-staining as compared with untreated cells (Figures 1(b) and 1(c)). 

### 3.2. Antioxidative Effects of PCS Extract on Hepatocytes

**Figure 2:**
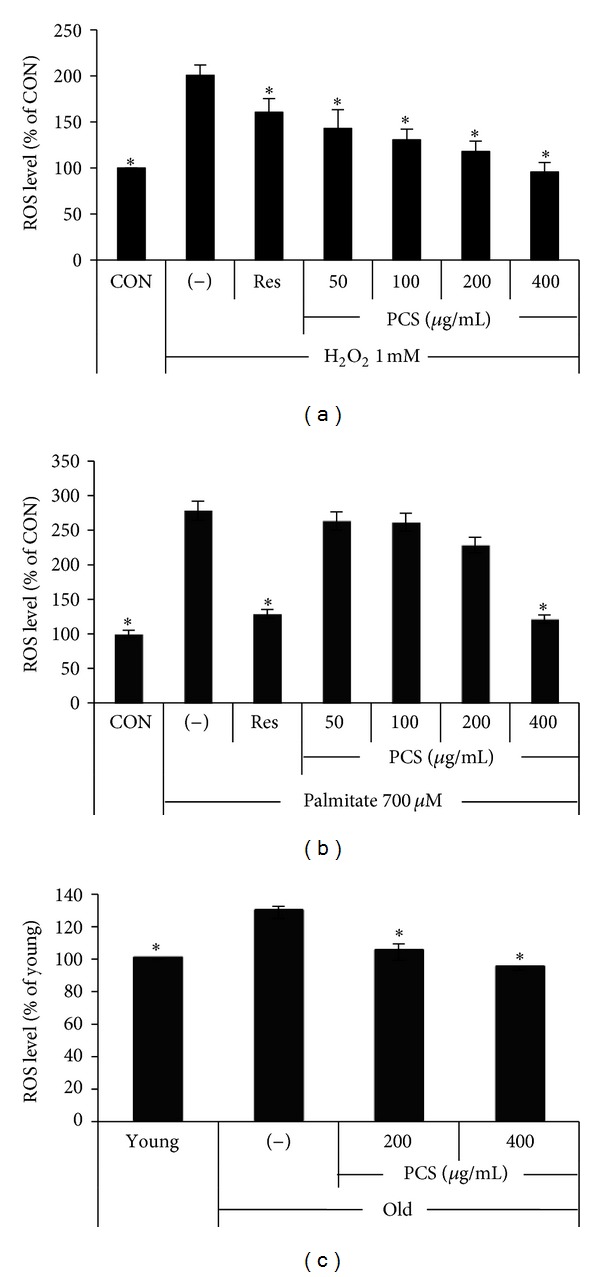
Antioxidant effects of the PCS extract on hepatocytes. (a) HepG2 cells were treated without (−) or with various doses of the PCS extract for 24 h, and 1 mM H_2_O_2_ was added for the last 3 h. *P < 0.05 versus (−)/H_2_O_2_. (b) HepG2 cells were treated without (−) or with various doses of the PCS extracts and 700 *μ*M palmitate for 24 h. *P < 0.05 versus (−)/palmitate. (c) Primary hepatocytes were isolated from young (2 months old) and old (20 months old) mice. After 24 h of treatment without (−) or with the PCS extract, ROS levels were measured. *P < 0.05 versus (−)/old. CON, treatment with nothing. Resveratrol (Res, 50 *μ*M) was used as a positive control.

To examine whether PCS extract has free radical-scavenging effects, we measured the intracellular ROS level in HepG2 cells after treatment with H_2_O_2_ or palmitate in the presence or absence of PCS extract. PCS extract significantly reduced the ROS level induced by H_2_O_2_ or palmitate in a dose-dependent manner (Figures 2(a) and 2(b)). ROS production was significantly lower in untreated hepatocytes of young mice (2 months old) compared with old mice (20 months old), and PCS extract significantly reduced the ROS level in hepatocytes of aged mice to levels comparable with young mice (Figure 2(c)). 

**Figure 3:**
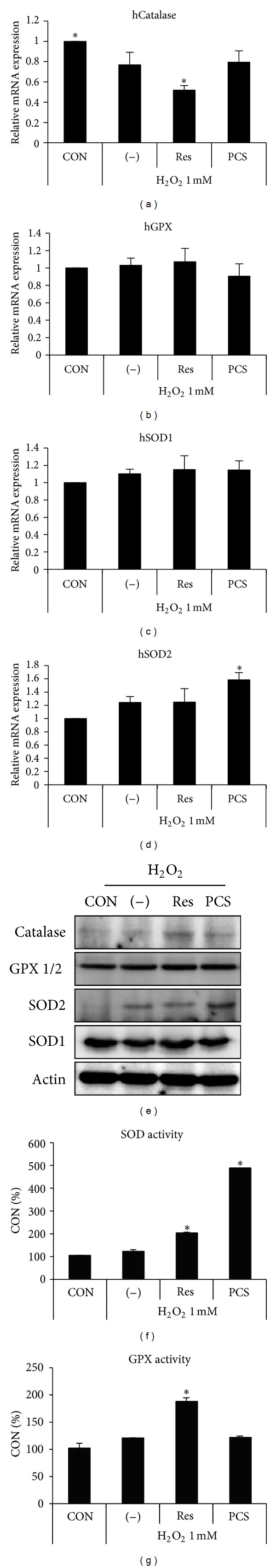
Antioxidant mechanism of the PCS extract on hepatocytes. HepG2 cells were treated with (200 *μ*g/mL) of the PCS extract for 24 h, and 1 mM H_2_O_2_ was added for the last 3 h. (a)–(d) The mRNA levels of antioxidation-related genes were analyzed by qRT-PCR. (e) Protein levels of antioxidation-related enzymes were analyzed using western blotting. (f) SOD activity. (g) GPX activity. *P < 0.05 versus (−)/H_2_O_2_. CON, treatment with nothing. Resveratrol (Res, 50 *μ*M) was used as a positive control.

We then examined the mRNA expression of genes related to antioxidant enzymes in H_2_O_2_-treated HepG2 cells by PCS extract treatment. Only SOD2 mRNA was significantly increased by PCS extract treatment (Figures 3(a)–3(d)). Similarly, PCS extract treatment increased the SOD2 protein (Figure 3(e)). When we examined the activity of antioxidant enzymes, we found that PCS extract significantly increased SOD activity (Figure 3(f)). However, the GPX activity was not changed (Figure 3(g)). These results suggest that the PCS extract increased the expression level of mRNA and protein of SOD2, subsequently increasing the activity of SOD. 

### 3.3. Protective Effect of PCS Extracts on H_**2**_O_**2**_-Induced Mitochondrial Dysfunction in Hepatocytes

**Figure 4:**
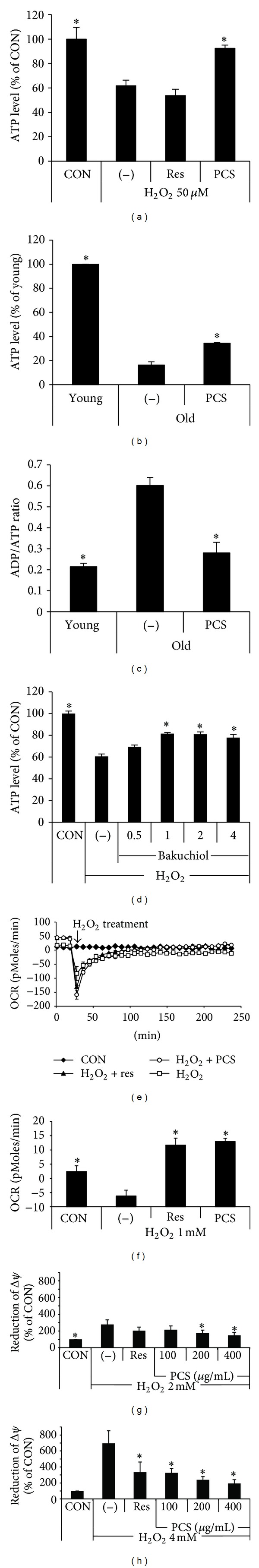
Effects of the PCS extract on H_2_O_2_-induced mitochondria dysfunction in hepatocytes. (a) HepG2 cells were treated with 50 *μ*M H_2_O_2_ for 2 h. After H_2_O_2_, 500 *μ*g/mL of the PCS extract was added and cells were incubated for 24 h. ATP levels and the ADP/ATP ratio were measured. *P < 0.05 versus (−)/H_2_O_2_. (b)-(c) Primary hepatocytes were isolated from young (2 months old) and old (20 months old) mice. Cells were treated without (−) or with 400 *μ*g/mL of PCS extract for 24 h, and the ATP levels (b) and ADP/ATP ratios (c) were measured. *P < 0.05 versus (−)/old. (d) HepG2 cells were treated with 50 *μ*M H_2_O_2_ for 2 h. After H_2_O_2_, 0.5–4.0 *μ*g/mL of bakuchiol was added and the cells were incubated for 24 h. The ATP levels were measured. *P < 0.05 versus (−)/H_2_O_2_. (e) HepG2 cells were cultured on Seahorse XF-24 plates. After overnight incubation, cells were treated without (−) or with 200 *μ*g/mL of the PCS extract for 20 h, and H_2_O_2_ was added during the OCR measurement. The OCR was automatically calculated and recorded by the Seahorse XF-24. (f) The OCR of the end of measurement time. *P < 0.05 versus (−)/H_2_O_2_. (g)-(h) HepG2 cells were treated without (−) or with the PCS extract for 24 h, and 2 (g) or 4 (h) mM H_2_O_2_ was added for the last 6 h. The reduction in mitochondrial membrane potential was determined using a mitochondrial membrane potential assay kit (*P < 0.05 versus (−)/H_2_O_2_. CON, no treatment. Resveratrol (Res, 50 *μ*M) was used as a positive control.

To determine whether PCS extract treatment affects mitochondrial function, we first measured ATP production in HepG2 cells after treatment with H_2_O_2_. H_2_O_2_ treatment significantly decreased the ATP level compared with untreated control cells, and PCS extract treatment significantly reversed this decreased ATP level (Figure 4(a)). In addition, PCS extract treatment increased the ATP level in hepatocytes of old mice, which was lower compared with that of young mice (Figure 4(b)). In parallel, the ADP/ATP ratio of untreated hepatocytes from old mice was increased as compared with young mice, and PCS extract treatment reduced the ADP/ATP ratio (Figure 4(c)). Treatment with bakuchiol, which is a major component of the PCS extract, also recovered the reduced ATP level by H_2_O_2_ treatment (Figure 4(d)).

To clarify the effect of improved mitochondrial function by PCS extract, OCR was measured. We found that H_2_O_2_ treatment significantly reduced the OCR measured over 250 min, and PCS extract treatment recovered the OCR, and the recovery was faster in PCS extract-treated HepG2 cells than in untreated cells (Figures 4(e) and 4(f)).

As mitochondrial function is correlated with the mitochondrial membrane potential (Δ*ψ*), we measured mitochondrial membrane potential reduction. H_2_O_2_ treatment increased the reduction in the mitochondrial membrane potential, and PCS extract treatment inhibited this increase (Figures 4(g) and 4(h)). These results indicate that PCS extract treatment improved mitochondrial function and protected the mitochondria from oxidative stress.

### 3.4. Improvement of Mitochondrial Biogenesis by PCS Extract in H_**2**_O_**2**_-Treated HepG2 Cells

**Figure 5:**
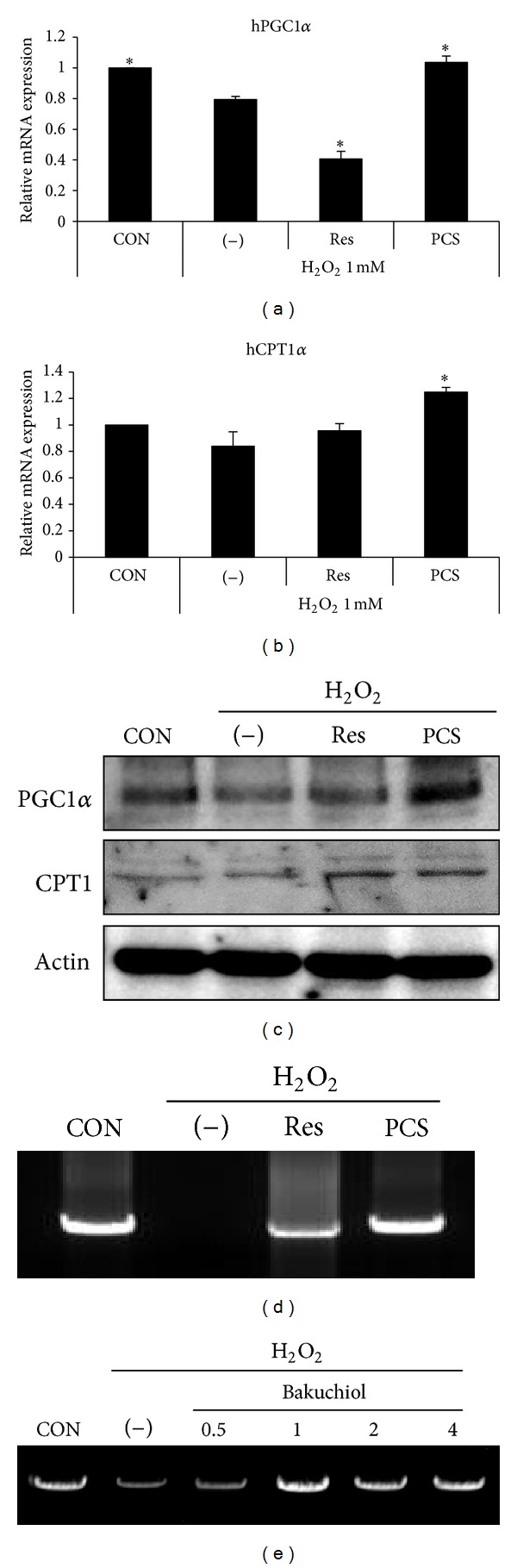
Effects of the PCS extract on mitochondrial biogenesis in H_2_O_2_-treated HepG2 cells. (a)–(d) HepG2 cells were treated without (−) or with 200 *μ*g/mL of the PCS extract for 24 h, and 1 mM H_2_O_2_ was added during the last 3 h. The mRNA levels of hPGC1*α* (a) and hCPT1*α* (b) and protein levels (c) were measured. (d) Genomic DNA was isolated from HepG2 cells. Long PCR of the mitochondrial genome was carried out. (e) HepG2 cells were treated without (−) or with 0.5–4 *μ*g/mL of bakuchiol for 24 h, and 1 mM H_2_O_2_ was added during the last 3 h. Genomic DNA was isolated from HepG2 cells. Long PCR of the mitochondrial genome was carried out. CON, treatment with nothing. Resveratrol (Res, 50 *μ*M) was used as a positive control.

We then examined mRNA and protein expression of PGC1*α*, which is a key regulator of mitochondrial biogenesis. Both the mRNA and protein levels of PGC1*α* were decreased in H_2_O_2_-treated cells as compared with the control cells, and this was reversed by PCS extract treatment (Figures 5(a) and 5(c)). The mRNA and protein levels of CPT1, which is an indicator of mitochondrial function, were also increased by PCS extract treatment (Figures 5(b) and 5(c)).

To verify the mitochondrial protective effects of PCS extract, we applied long PCR amplification to half of the mitochondrial genome. Untreated HepG2 cells showed high intensity of the 8.7 kb band, which was substantially decreased in the H_2_O_2_-treated cells (Figure 5(d)). PCS extract treatment recovered the 8.7 kb band intensity; this effect was stronger as compared with resveratrol treatment (Figure 5(d)). Bakuchiol, which is a major component of the PCS extract, also recovered the disappearance of mitochondrial DNA by H_2_O_2_ treatment (Figure 5(e)). These results indicate that the PCS extract has protective effects on mitochondrial DNA against oxidative stress, and bakuchiol has a main role in the protective effect on hepatic mitochondria.

## 4. Discussion

Aging is a degenerative process that is characterized by a gradual functional decline of all organ systems and increased susceptibility to diseases. Mitochondrial damage and mitochondrial alterations, including an increase of ROS generation and decrease of mitochondrial oxidative phosphorylation, occur during the aging process [8, 20]. Therefore, modulation of these age-associated mitochondrial changes may slow the aging process and prevent or delay age-related diseases. PCS has been used traditionally as a medicine in Asia and are known to have antioxidant activity [21–23]. In particular, a component of PCS has liver detoxifying and hepatoprotective effects [16, 17, 24]. In this study, we investigated the protective effects of PCS extracts in mitochondrial dysfunction induced in cultured hepatocytes by H_2_O_2_ or in primary hepatocytes from old mice.

Cellular senescence can be characterized by the expression of specific markers such as p16^INK4a^ and SA-*β*-gal [25, 26]. We examined the PCS extract for antisenescence effects by analyzing the mRNA expression of p16^INK4a^ and SA-*β*-gal expression. PCS extract treatment resulted in the most pronounced reversal of the age-related increase in p16^INK4a^ and SA-*β*-gal in HDF cells, suggesting that PCS extract might have an antisenescence effect.

As ROS are known to play a central role in mediating various metabolic disorders related to aging, inhibiting ROS production and enhancing ROS scavenging may be useful for treating aging and age-related metabolic disorders [3, 7, 27]. Therefore, we checked whether PCS extract has any inhibitory effects on ROS generation. Treatment with PCS extract significantly reduced H_2_O_2_- or palmitate-induced ROS generation. In addition, PCS extract significantly scavenged intracellular ROS in the primary hepatocytes of old mice. These results suggest that PCS extract is effective for protecting hepatocytes from ROS toxicity. 

ROS removal is regulated by many antioxidant enzymes, including SOD1, SOD2, GPX, and catalase [28], and overexpression of SOD2 protects against alcohol-induced liver injury [29]. In our study, both the mRNA and protein level of SOD2, which is a mitochondrial scavenging enzyme, were increased by PCS extract treatment. Several studies reported that resveratrol has an antioxidative effect through the activation of antioxidant enzymes including SOD, GPX, and catalase [30–32]. In our study, treatment of the resveratrol, which was used as a positive control, showed an increase of SOD and GPX activity, and increase of the catalase protein level [32]. But PCS extract treatment significantly increased the activity of SOD in HepG2 cells treated with H_2_O_2_. As PCS extract treatment particularly increased SOD2 mRNA expression, we consider that PCS extract has an antioxidative effect through mitochondrial improvement. 

As ATP generation is an essential function in mitochondria, we examined the effect of PCS extract on ATP synthesis. PCS extract and bakuchiol treatment increased ATP synthesis, which was reduced by H_2_O_2_ treatment in the hepatocytes of old mice. In parallel, OCR was increased by PCS extract treatment. We then examined the integrity of the mitochondrial membrane structure, which is involved in ATP energy production and mitochondrial function [33]. PCS extract treatment recovered the reduced mitochondrial potential induced by oxidative stress. These results indicate that PCS extract stimulates mitochondrial respiration and restores mitochondrial energy metabolism.

CPT1 and PGC1 are important mitochondrial proteins. CPT1 is associated with the mitochondrial outer membrane and regulates energy production from the main oxidative substrates [34]. PGC1*α* controls many aspects of oxidative metabolism, including mitochondrial biogenesis, and respiration [35]. Reduction of PGC1 in animals by either genetic knockout or RNAi confers hypersensitivity to death from oxidative stress [36]. In our study, both the mRNA and protein level of CPT1 and PGC1*α* were increased by the PCS extract. As well, mitochondrial genome damage was protected by the PCS extract and bakuchiol treatment. These results suggest that PCS extract treatment protects against hepatocyte damage by stimulating mitochondrial biogenesis and, bakuchiol is one major component of the hepatic mitochondrial protective effect of the PCS extract.

Many studies have established that oxidative stress and mitochondrial dysfunction are two central factors contributing to the aging process. Mitochondrial size, numbers, and function are altered in aging [37–39]. As the liver has many mitochondria and plays an important role in the whole body metabolism process, hepatic mitochondrial biogenesis and improvement of mitochondrial function are important to the whole body metabolism, as well as the hepatic metabolism for the aging process. Therefore, a better understanding of the response to oxidative stress and mitochondrial regulation in hepatocytes will reveal new therapeutic targets for age-associated degenerative diseases. PCS extract may be a beneficial plant-based dietary component to counteract oxidative stress-induced disease or aging.

## Figures and Tables

**Table 1:** Primers used for quantitative real-time PCR.

Gene	Forward/reverse primers
p16^INK4a^	5′-GAAGGTCCCTCAGACATCCCC
	5′-CCCTGTAGGACCTTCGGTGAC
Cyclophilin	5′-TGCCATCGCCAAGGAGTAG
	5′-TGCACAGACGGTCACTCAAA
Catalase	5′-TTTCCCAGGAAGATCCTGAC
	5′-ACCTTGGTGAGATCGAATGG
GPX	5′-AGAATGTGGCGTCCCTCTGA
	5′-CAGCTCGTTCATCTGGGTGTAG
SOD1	5′-GGTCCTCACTTTAATCCTCTAT
	5′-CATCTTTGTCAGCAGTCACATT
SOD2	5′-TTCTGGACAAACCTCAGCCC
	5′-AGTTTGATGGCTTCCAGCA
PGC1*α*	5′-GTGAAGACCAGCCTCTTTGC
	5′-TCACGTCTCCATCTATCAGC
CPT1*α*	5′-CGTCTTTTGGGATCCACGATT
	5′-TGTGCTGGATGGTGTCTGTCTC

## Abbreviations

CPT1: Carnitine palmitoyltransferase 1
DMEM: Dulbecco's Modified Eagle's Medium
GPX 1/2: Glutathione peroxidase 1/2
HDF: Human diploid fibroblasts
OCR: Oxygen consumption rate
PCS: *Psoralea corylifolia* seed
PGC1*α*: Peroxisome proliferator-activated receptor *γ* coactivator 1*α*
ROS: Reactive oxygen species
SA-*β*-gal: Senescence-associated *β*-galactosidase
SOD: Superoxide dismutase.
